# Supplementary material for: Gene mobility elements mediate cell type specific genome organization and radial gene movement in vivo
Source: bioRxiv. 2024 Dec 1:2024.11.30.626181. Preprint. [Version 1] doi: 10.1101/2024.11.30.626181 (PMC11623685; doi:10.1101/2024.11.30.626181)
Supplement: 1 [file NIHPP2024.11.30.626181V1-supplement-1.pdf]

bioRxiv preprint doi: https://doi.org/10.1101/2024.11.30.626181; this version posted December 1, 2024. The copyright holder for this preprint (which was not certified by peer review) is the author/funder, who has granted bioRxiv a license to display the preprint in perpetuity. It is made available under aCC-BY 4.0 International license.

Supplementary figures to Lucas et al., Gene mobility elements mediate cell type specific genome organization and radial gene movement *in vivo*

bioRxiv preprint doi: https://doi.org/10.1101/2024.11.30.626181; this version posted December 1, 2024. The copyright holder for this preprint (which was not certified by peer review) is the author/funder, who has granted bioRxiv a license to display the preprint in perpetuity. It is made available under aCC-BY 4.0 International license.

Supp Figure 1

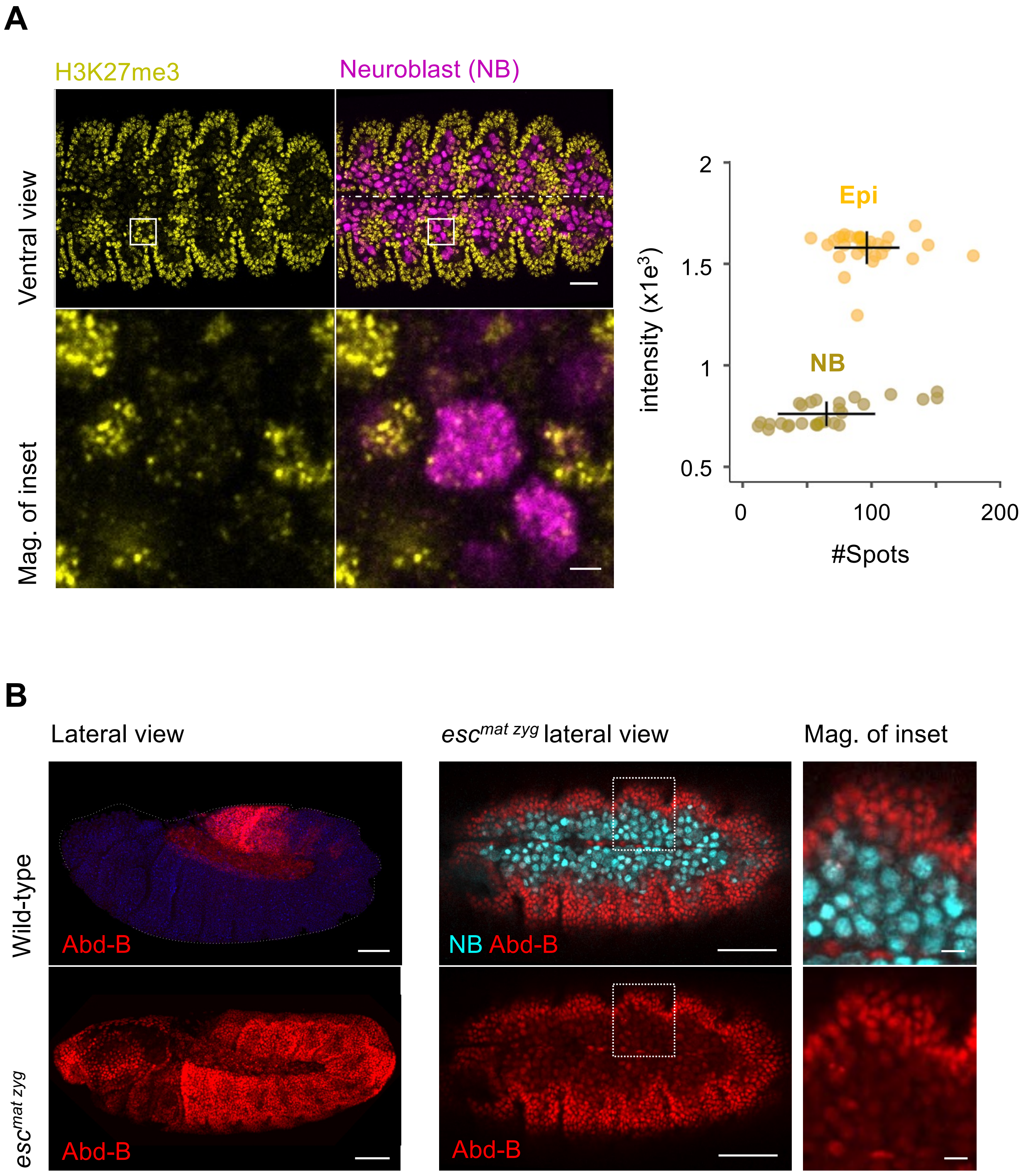

bioRxiv preprint doi: https://doi.org/10.1101/2024.11.30.626181; this version posted December 1, 2024. The copyright holder for this preprint (which was not certified by peer review) is the author/funder, who has granted bioRxiv a license to display the preprint in perpetuity. It is made available under aCC-BY 4.0 International license.

Supplemental Figure 1 (Supplemental to Figure 1):

A. H3K27me3 signal is decreased in neuroblast: *Left*, Immunostaining of the Polycomb repressive complex 2 epigenetic mark H3K27me3 in stage 14 embryos. Ventral single Z-plane showing pan-neuroblast Worniu marker (Wor) in magenta and the H3K27me3 marks in yellow. The Embryonic midline is shown as a dashed white line. Scale bar = 20µm, scale bar of close up = 2µm. *Right*, scatter plot quantification of H3K27me3 foci in neuroblast vs non neuroblast showing the background normalized intensity of H3K27me3 staining signal in function of the number of H3K27me3 detected foci (#Spots). Standard deviations are shown as vertical and horizontal black lines and average at the intersection.

B. Lack of Abd-B derepression in PRC2 mutant embryo: Immunostaining of wild-type and maternal and zygotic mutant of the PRC2 subunit extra-sex-combs, *esc*. *Left*, Side views of wild-type and *esc* mutant embryos stained for Abd-B in red, and DAPI in blue (wild-type only). Scale bars = 50µm. *Middle*, Single Z-plan of ventral view of the *esc* mutant embryos stained for Abd-B in red and pan-neuroblast marker Wor in cyan. Scale bar = 50µm. *Right*, magnification of the insets from middle panels, Scale bar = 20µm.

bioRxiv preprint doi: https://doi.org/10.1101/2024.11.30.626181; this version posted December 1, 2024. The copyright holder for this preprint (which was not certified by peer review) is the author/funder, who has granted bioRxiv a license to display the preprint in perpetuity. It is made available under aCC-BY 4.0 International license.

Supp Figure 2

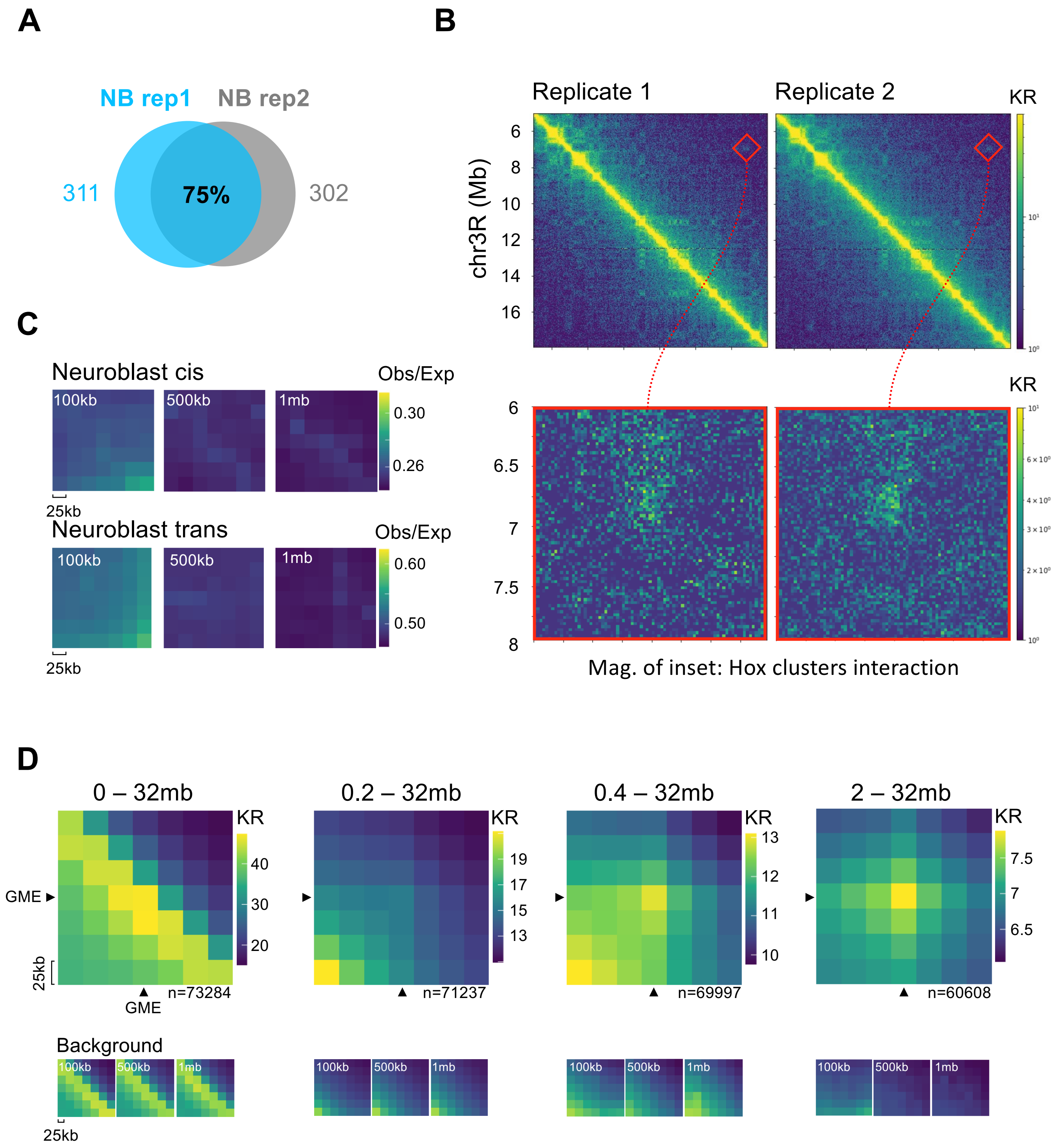

bioRxiv preprint doi: https://doi.org/10.1101/2024.11.30.626181; this version posted December 1, 2024. The copyright holder for this preprint (which was not certified by peer review) is the author/funder, who has granted bioRxiv a license to display the preprint in perpetuity. It is made available under aCC-BY 4.0 International license.

Supplemental Figure 2 (Supplemental to Figure 1):

A. Strong TAD borders overlap among Hi-C neuroblast replicates: Venn diagram showing the overlap of detected TAD borders in neuroblast from two Hi-C biological replicates 25 kb resolution of embryonic neuroblast stage 10. Hi-C maps were normalized and KR-corrected.

B. Hox clusters interactions are detected in neuroblast Hi-C replicates: *Top*, 25 kb resolution Hi-C matrices from two biological replicates of neuroblast embryonic stage 10 spanning ANT-C and BX-C Hox gene clusters on chromosome 3R (highlighted by the red squares). *Bottom*, magnification of Hox cluster interaction. Hi-C maps were normalized and KR-corrected and shown as log1p scale.

C. Background at GME-GME interaction: Average submatrices, sampled at distances of 100 kb, 500 kb, and 1 Mb from the GME-GME cis or trans interaction in neuroblast. 25 kb resolution Hi-C matrices were KR-correct and the Observed/Expected (O/E) is shown.

D. GME-GME cis interactions are detected in neuroblasts : Average submatrices of all possible GME-GME cis interactions in neuroblasts at various distance ranges. GME- containing Hi-C bins are pointed by black arrows. The total number of GME-GME interactions (n=) are shown and the background average submatrices, sampled at distances of 100 kb, 500 kb, and 1 Mb from GME-GME interactions are shown below each GME-GME average Hi-C submatrices. 25 kb resolution Hi-C matrices were KR-correct.

bioRxiv preprint doi: https://doi.org/10.1101/2024.11.30.626181; this version posted December 1, 2024. The copyright holder for this preprint (which was not certified by peer review) is the author/funder, who has granted bioRxiv a license to display the preprint in perpetuity. It is made available under aCC-BY 4.0 International license.

Supp Figure 3

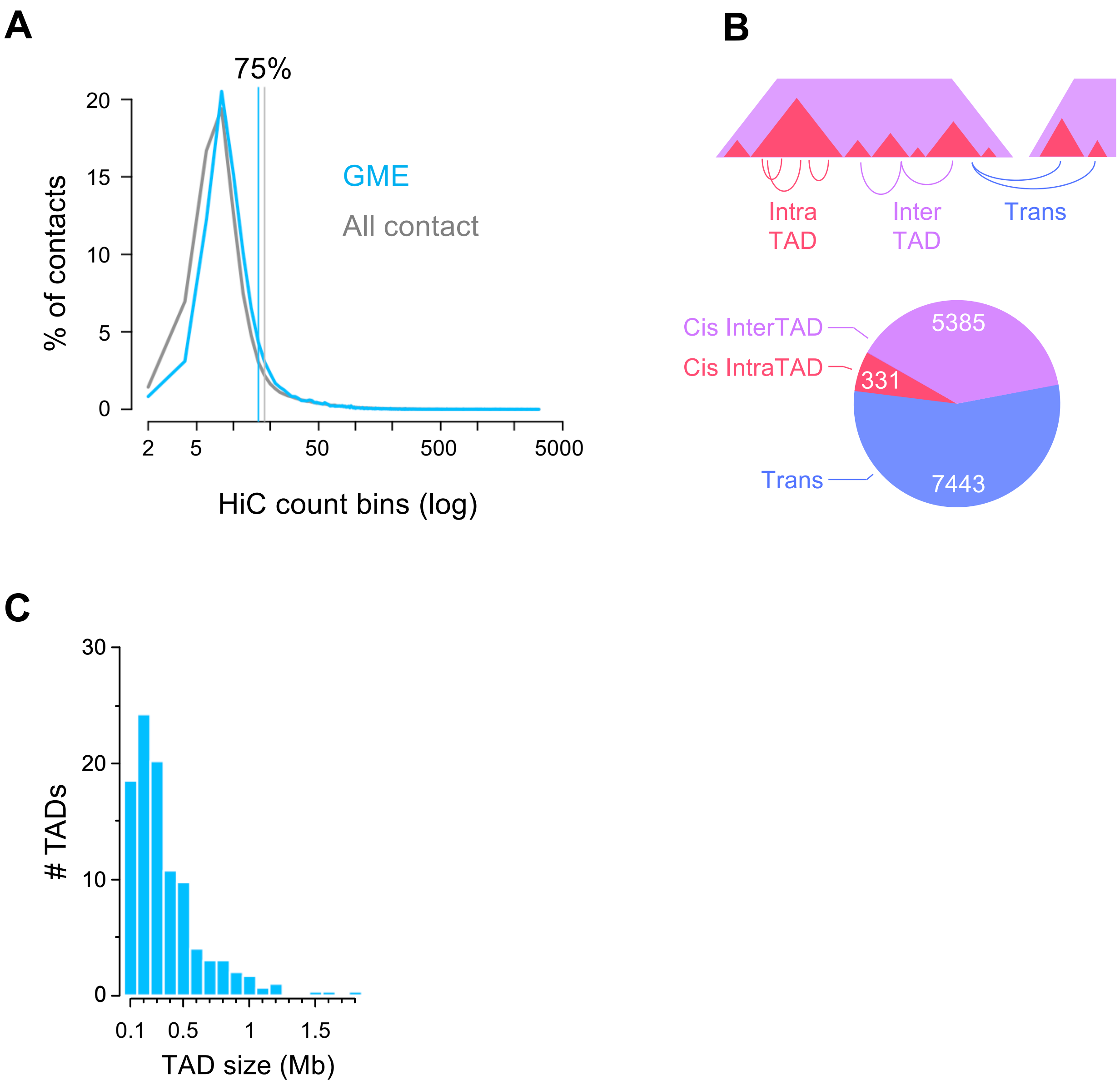

bioRxiv preprint doi: https://doi.org/10.1101/2024.11.30.626181; this version posted December 1, 2024. The copyright holder for this preprint (which was not certified by peer review) is the author/funder, who has granted bioRxiv a license to display the preprint in perpetuity. It is made available under aCC-BY 4.0 International license.

Supplement Figure 3 (Supplemental to Figure 2):

A. GME significant contacts and all significant contacts Hi-C count: Distribution of the proportion of significant contacts in function of Hi-C count (KR-corrected) shown on a log scale. Horizontal lines mark the 75th percentile of the distributions.

B. GME-GME contact detection: *Top,* Schematic representation of contact types detected in the Hi-C data. Cis contacts connect regions within the same chromosome: cis, intra contacts occur within the same TAD, while cis, inter contacts span across different TADs on the same chromosome arm. Trans contacts connect regions from different chromosomes or chromosome arms. *Bottom*, Circular diagram showing the number of significant contacts per type.

C. TAD size distribution: Distribution of the size of detected TAD (n=297) genome wide (mean ~400 kb, median of ~350 kb).

bioRxiv preprint doi: https://doi.org/10.1101/2024.11.30.626181; this version posted December 1, 2024. The copyright holder for this preprint (which was not certified by peer review) is the author/funder, who has granted bioRxiv a license to display the preprint in perpetuity. It is made available under aCC-BY 4.0 International license.

Supp Figure 4

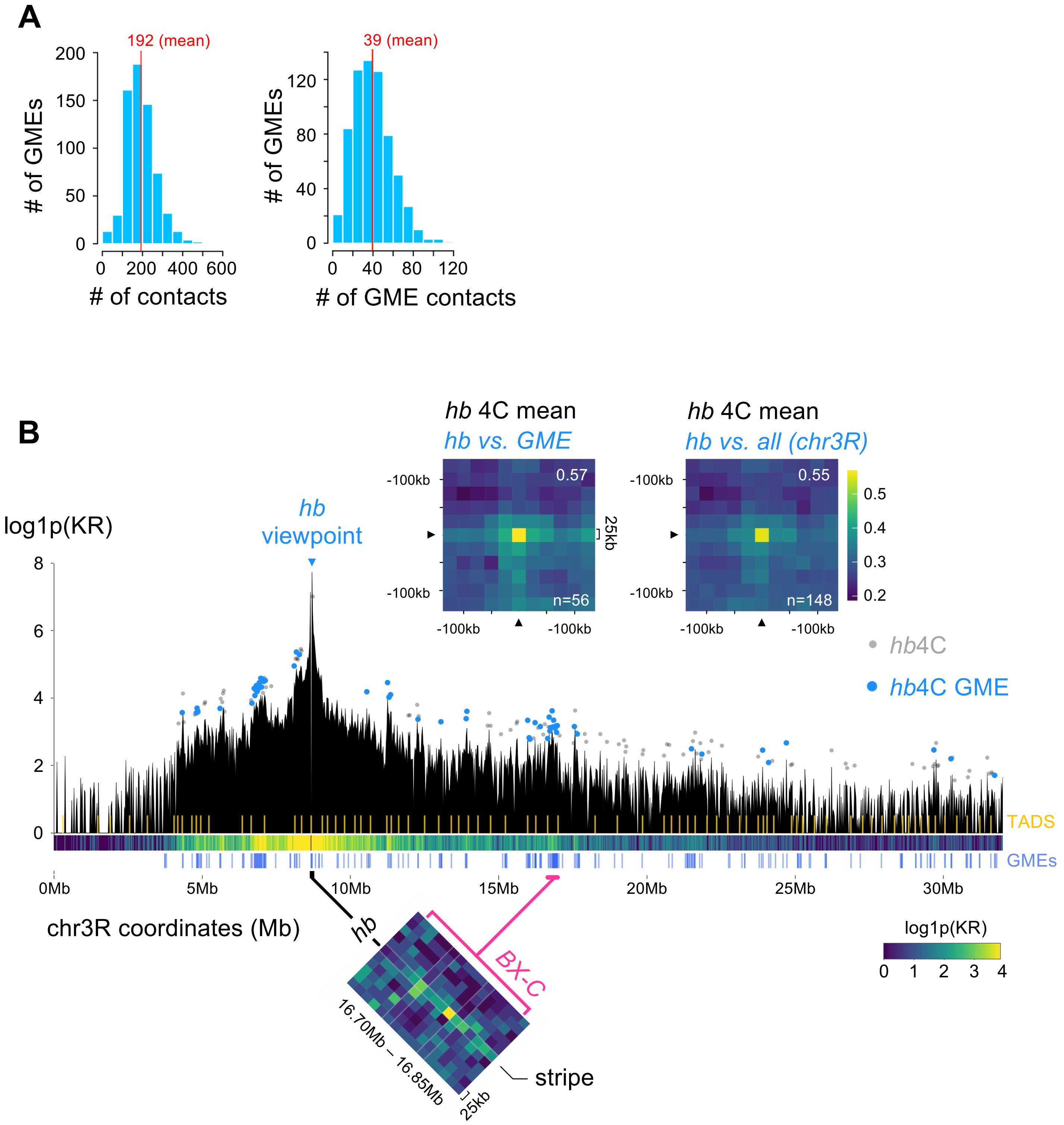

bioRxiv preprint doi: https://doi.org/10.1101/2024.11.30.626181; this version posted December 1, 2024. The copyright holder for this preprint (which was not certified by peer review) is the author/funder, who has granted bioRxiv a license to display the preprint in perpetuity. It is made available under aCC-BY 4.0 International license.

Supplemental Figure 4: (Supplemental to Figure 2):

A. GME Contact Statistics: *Left***,** Distribution of significant contacts from the virtual 4C of each GME viewpoint. *Right***,** Distribution of significant GME-GME contacts within the virtual 4C of each GME viewpoint.

B. *hunchback* 4C: Virtual 4C analysis of the *hb* GME on chromosome 3R from KR-correct neuroblasts Hi-C matrix at 25 kb resolution. Contact types: Contacts involving GMEs are shown in blue, while non-GME contacts are depicted in grey. Yellow lines indicate Topologically Associating Domain (TAD) borders. GMEs positions are shown as blue vertical bars. *Top,* Average Hi-C submatrices of all the 4C contacts involving the *hb* GME and either other GMEs (*top-left*) or all other contacts (*top-right*) . Hi-C bins involved in the significant contacts are pointed by black arrows. Total number of contacts are shown in white. *Bottom,* Zoom-in of the contact between the GME of *hb* and the BX-C Hox cluster GMEs. KR-corrected and Observed/Expected values are shown.

bioRxiv preprint doi: https://doi.org/10.1101/2024.11.30.626181; this version posted December 1, 2024. The copyright holder for this preprint (which was not certified by peer review) is the author/funder, who has granted bioRxiv a license to display the preprint in perpetuity. It is made available under aCC-BY 4.0 International license.

Supp Figure 5

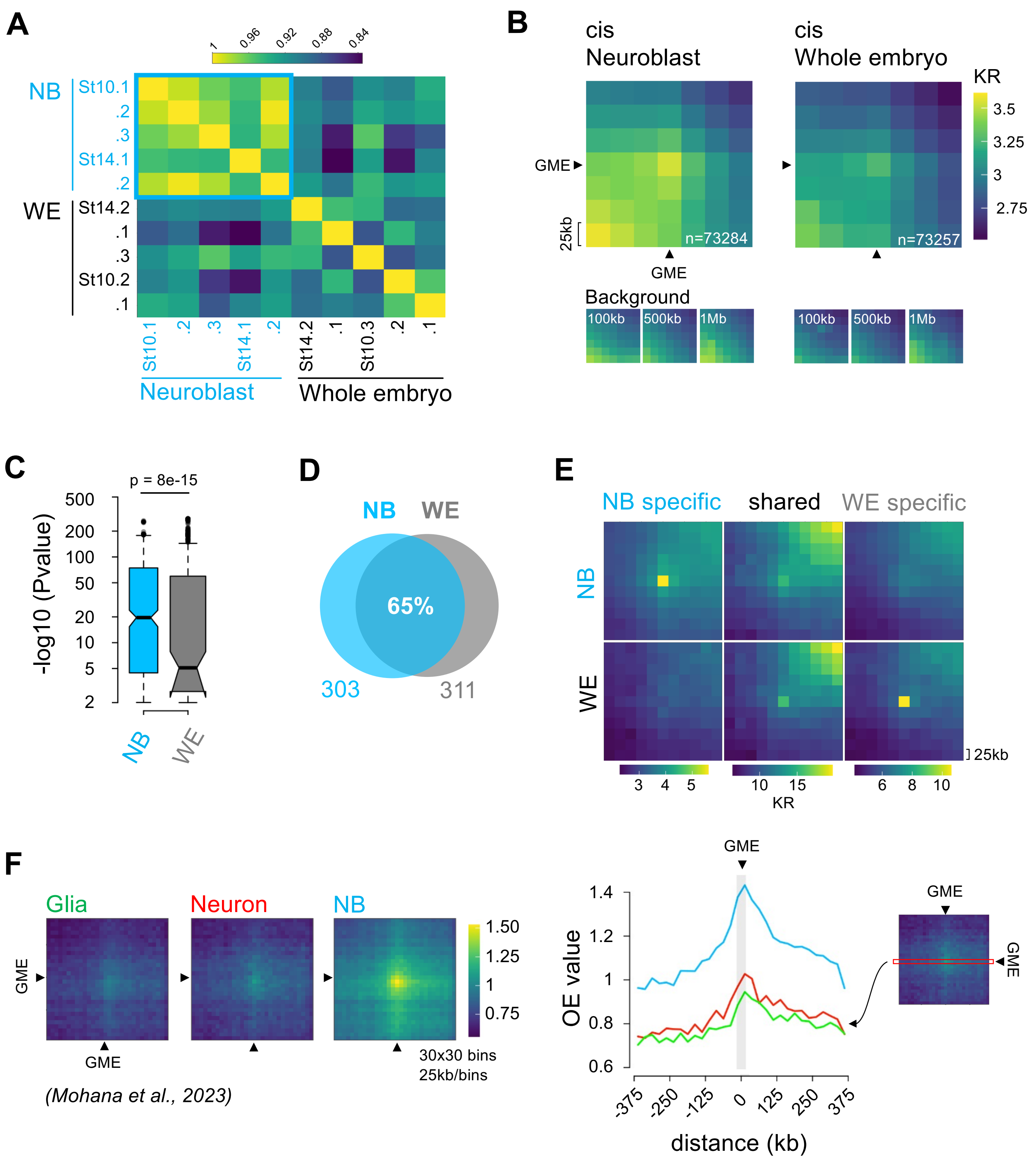

bioRxiv preprint doi: https://doi.org/10.1101/2024.11.30.626181; this version posted December 1, 2024. The copyright holder for this preprint (which was not certified by peer review) is the author/funder, who has granted bioRxiv a license to display the preprint in perpetuity. It is made available under aCC-BY 4.0 International license.

Supplemental Figure 5 (Supplemental to Figure 3):

A. Hi-C sample correlation: Correlation heatmap showing HiCREP *scc* scores for neuroblast (blue labels) and whole embryo (black labels) Hi-C replicates at 25 kb resolution. Hi-C data were unnormalized and uncorrected.

B. GME-GME interactions are stronger in neuroblast than whole embryo: *Top*: Average Hi-C submatrices of all possible GME-GME cis interactions in neuroblast and whole embryo cells. 25 kb resolution HiC matrices were normalized and KR-corrected. GME-containing Hi-C bins are pointed by black arrows. Total number of GME-GME interactions are shown in white. *Bottom:* Average submatrices, sampled at distances of 100 kb, 500 kb, and 1 Mb from the GME-GME interaction.

C. GME-GME contacts are more significant in neuroblast than whole embryo: Distribution of significant cis contacts p-values in neuroblast and whole embryo. Wilcoxon test was used for significance.

D. TAD borders overlap: Venn diagram showing the overlap of detected TAD borders in neuroblast and whole embryo at 25 kb Hi-C resolution. Hi-C maps were normalized and KR-corrected.

E. Neuroblasts make cell-type specific GME-GME contacts: Average Hi-C submatrice of neuroblast specific, whole embryo specific and shared significant GME-GME contacts in neuroblast and whole embryo. Hi-C data were normalized and KR-corrected values were displayed. Analyzed contacts were thresholded by distances ranging from 0.4 Mb to 32 Mb.

F. GME-GME contacts are stronger in neuroblast compared to other CNS cell types: Average Hi-C submatrice of neuroblast GME-GME significant contacts in glia, neurons and neuroblast from Mohana et al., 2023 (GSE214707). 25 kb resolution Hi-C data were normalized, KR-corrected and observed/expected values were displayed.

bioRxiv preprint doi: https://doi.org/10.1101/2024.11.30.626181; this version posted December 1, 2024. The copyright holder for this preprint (which was not certified by peer review) is the author/funder, who has granted bioRxiv a license to display the preprint in perpetuity. It is made available under aCC-BY 4.0 International license.

Supp Figure 6

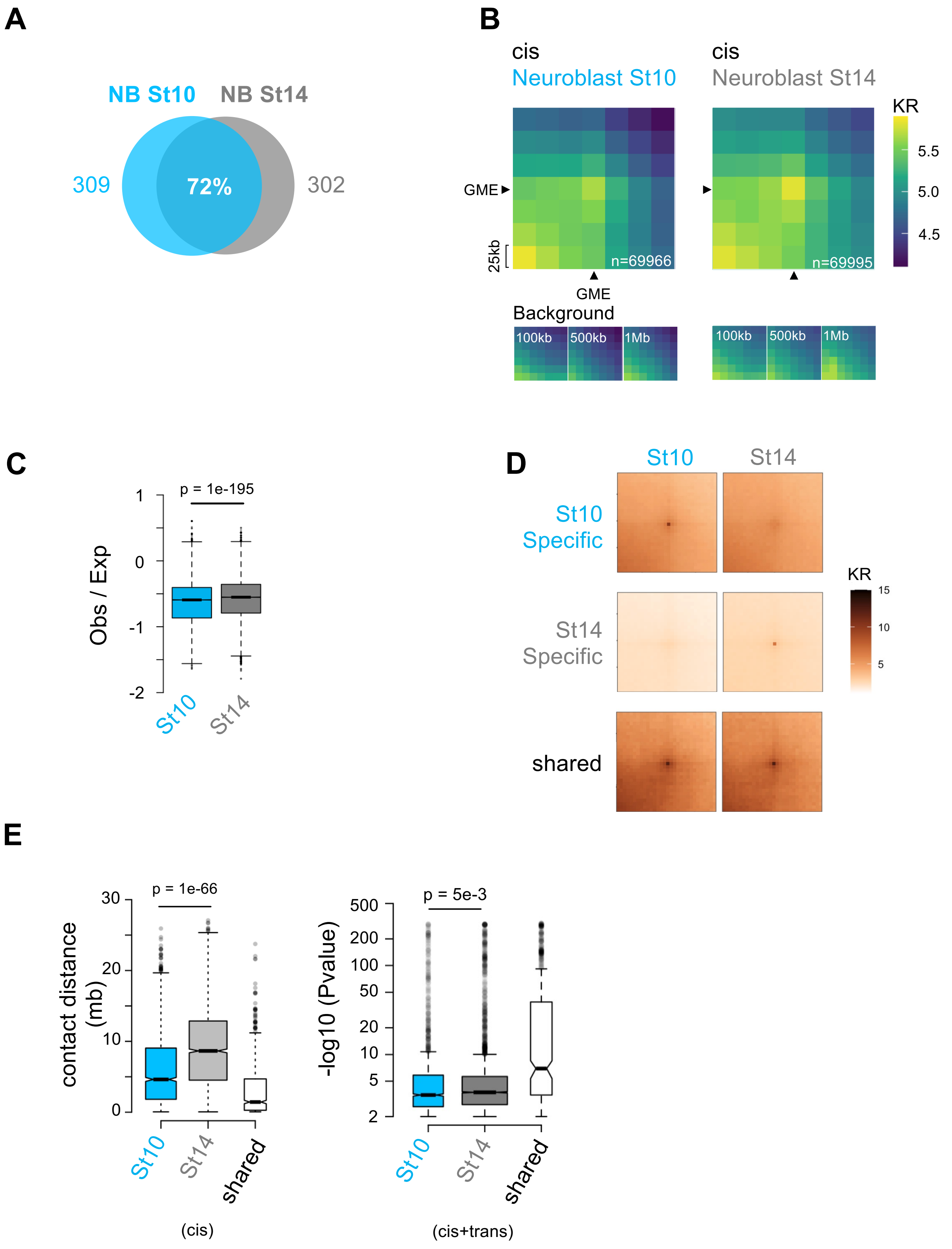

bioRxiv preprint doi: https://doi.org/10.1101/2024.11.30.626181; this version posted December 1, 2024. The copyright holder for this preprint (which was not certified by peer review) is the author/funder, who has granted bioRxiv a license to display the preprint in perpetuity. It is made available under aCC-BY 4.0 International license.

Supplemental Figure 6 (Supplemental to Figure 4):

A. Neuroblasts TAD border overlap: Venn diagram showing the overlap of detected TAD borders in neuroblast stage 10 and14 at 25 kb Hi-C resolution. Hi-C maps were normalized and KR-corrected.

B. Cis GME-GME interaction over time: *Top*: Average Hi-C submatrices of all possible GME-GME cis interactions in neuroblast stage 10 and 14. 25 kb resolution HiC matrices were normalized and KR-corrected values were displayed. GME-containing Hi-C bins are pointed by black arrows. Total number of GME-GME interactions are shown in white. *Bottom:* Average submatrices, sampled at distances of 100 kb, 500 kb, and 1 Mb from the GME-GME interaction.

C. Strength of *cis* GME-GME interaction: Boxplot comparing the distribution of Hi-C Observed/Expected values of neuroblast stage 10 and stage 14. Stage 10: n=73253 interactions, including 12428 null. Stage 14: n=73282 interactions, including 8108 null interactions. Wilcoxon test was used for significance.

D. Stage specific GME contacts: Average Hi-C submatrice of neuroblast stage 10 specific, stage 14 specific and shared significant GME-GME contacts in neuroblast stage 10 and 14. Hi-C data were normalized and KR-corrected values were displayed. Analyzed contacts were thresholded by distance ranging from 0.4 Mb to 32 Mb.

E. Stage specific and shared GME-GME contact distance distribution and P-value: Boxplots comparing the distance (left) or the Pvalue (right) distribution of cis stage-specific and stage-shared GME-GME contacts. Wilcoxon test was used for significance.

bioRxiv preprint doi: https://doi.org/10.1101/2024.11.30.626181; this version posted December 1, 2024. The copyright holder for this preprint (which was not certified by peer review) is the author/funder, who has granted bioRxiv a license to display the preprint in perpetuity. It is made available under aCC-BY 4.0 International license.

Supp Figure 7

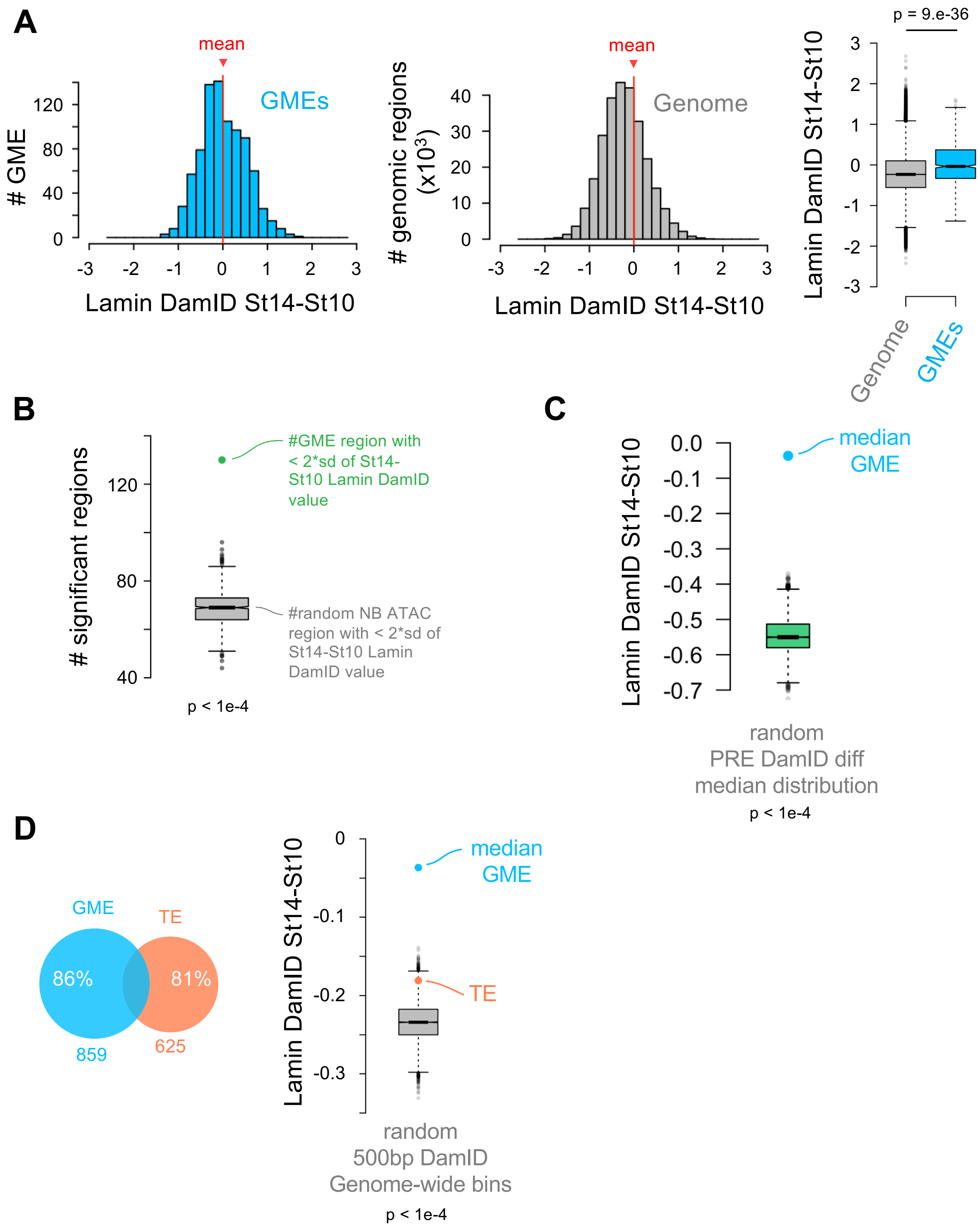

bioRxiv preprint doi: https://doi.org/10.1101/2024.11.30.626181; this version posted December 1, 2024. The copyright holder for this preprint (which was not certified by peer review) is the author/funder, who has granted bioRxiv a license to display the preprint in perpetuity. It is made available under aCC-BY 4.0 International license.

Supplemental Figure 7 (Supplemental to Figure 4):

A. Lamin DamID distribution at GMEs and genome-wide: Distribution of GMEs (Left) or whole genome (Middle) relative to their differential Lamin DamID signal (DamID stage 14 - stage 10); Means are displayed as red vertical lines and arrows. Right: Boxplot showing the distribution of the differential Lamin DamID signal genome-wide compared to GME regions. Wilcoxon test was used for significance.

B. Lamin DamID at GME vs non GME: Boxplot comparing the number of GME for which the differential DamID value is exceeding 2 standard deviations above the genome-wide average, green dot (see figure 4E) to the distribution of median of differential DamID value is exceeding 2 standard deviations above the genome-wide average from randomly sampled ATAC open sites excluding GME regions. 10000 random sampling of 859 ATAC open site were performed (bootstrap p-value < 1e-4). The black bar represents the median of the random distribution.

C. Lamin DamID at GME vs PREs: Boxplot comparing the median of the differential DamID of GMEs to that of the distribution of median from randomly sampled PREs. 10000 random sampling of 859 PREs were performed (bootstrap p-value < 1e-4). The black bar represents the median of the random distribution.

D. GME versus TE comparison: *Left*: Venn diagrams showing the overlap between GME and Tethering Elements (TE) from Batut et al., 2022. *Right*: Boxplot comparing the medians of the differential DamID of GMEs and TEs to that of the distribution of median from randomly sampled genome-wide 500bp regions. 10000 random sampling of 625 genome-wide regions were performed (bootstrap p-value < 1e-4). The black bar represents the median of the random distribution.

bioRxiv preprint doi: https://doi.org/10.1101/2024.11.30.626181; this version posted December 1, 2024. The copyright holder for this preprint (which was not certified by peer review) is the author/funder, who has granted bioRxiv a license to display the preprint in perpetuity. It is made available under aCC-BY 4.0 International license.

Sup Figure 8

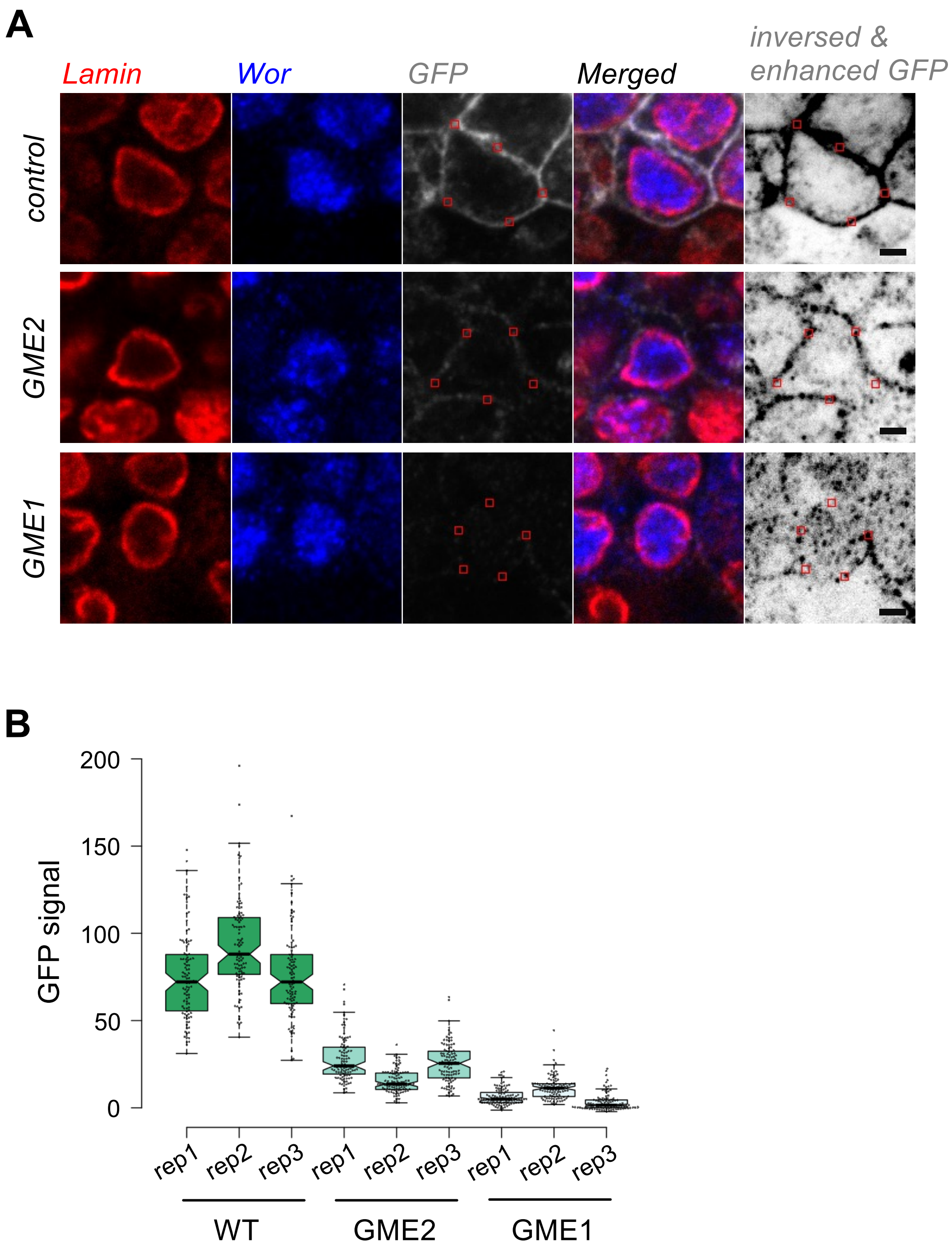

bioRxiv preprint doi: https://doi.org/10.1101/2024.11.30.626181; this version posted December 1, 2024. The copyright holder for this preprint (which was not certified by peer review) is the author/funder, who has granted bioRxiv a license to display the preprint in perpetuity. It is made available under aCC-BY 4.0 International license.

Supplemental Figure 8 (Supplemental to Figure 5):

A. Example of GFP quantification from mCD4 transgenes: Representative images of neuroblasts used for mCD4-GFP quantification, including Lamin (red), Worniu (blue), GFP (circled in red), a merged image, and an inverted merged image with enhanced GFP signal. The outlined workflow highlights steps for isolating GFP signals for analysis.

B. mCD4 transgene expression quantification across embryos: GFP signal intensity quantified on an 8-bit scale (0–255). For each embryo, GFP fluorescence from the dpn-mCD4-GFP transgene was sampled at 100 random positions along the neuroblast plasma membrane and at 50 random background positions (outside cells). Plasma membrane intensity was background-corrected by subtracting the average background signal. Scale bar: 2 μm.
